# Spatial distribution of inadequate meal frequency and its associated factors among children aged 6–23 months in Ethiopia: Multilevel and spatial analysis

**DOI:** 10.1371/journal.pone.0306646

**Published:** 2024-07-10

**Authors:** Tigabu Kidie Tesfie, Bekalu Endalew, Molla Yigzaw Birhanu, Aysheshim Belaineh Haimanot, Anteneh Lamesgen Mneneh, Muluye Gebrie Mengie, Elyas Melaku Mazengia, Mulat Belay Simegn, Muluken Chanie Agimas, Nebiyu Mekonnen Derseh, Girum Shibeshi Argaw, Werkneh Melkie Tilahun

**Affiliations:** 1 Department of Epidemiology and Biostatistics, Institute of Public Health, College of Medicine and Health Sciences, University of Gondar, Gondar, Ethiopia; 2 Department of Public Health, College of Medicine and Health Sciences, Debre Markos University, Debre Markos, Ethiopia; 3 Department of Nursing, College of Medicine & Health Sciences, Jigjiga University, Jigjiga, Ethiopia; University of Uyo, NIGERIA

## Abstract

**Introduction:**

More than two-third of global child death is occurred due to inappropriate feeding practice that happened during early childhood period. Evidence on meal frequency status among infant and young children at national level can be used to design appropriate interventions to improve the recommended feeding frequency. Therefore, this study was aimed to explore the spatial distribution and identify associated factors of inadequate meal frequency among children aged 6–23 months in Ethiopia.

**Methods:**

Secondary data analysis was conducted using the 2019 mini Ethiopian Demographic and Health Survey data. A total weighted sample of 1,532 children aged 6–23 months were included. To identify significant factors associated with of inadequate meal frequency, multilevel binary logistic regression model was fitted. Variables with p-value < 0.25 from the bi-variable model were exported to multivariable analysis. In the multivariable model, variables with p-value < 0.05 were declared as significantly associated factors and adjusted odds ratio (AOR) with its 95% confidence interval were reported. Multilevel models were compared using deviance and log-likelihood. Spatial analysis tools were utilized to visualize the distribution of inadequate meal frequency. Bernoulli model was fitted using SaTScan V.9.6 to identify most likely clusters and ArcGIS V.10.8 was used to map the hotspot areas. Ordinary least square and geographic weighted regression models were used and compared using information criteria and adjusted-R^2^. Local coefficients of factors associated with hotspots of inadequate meal frequency were mapped.

**Results:**

The prevalence of inadequate meal frequency was 47.03% (95% CI: 44.54%, 49.53%) in Ethiopia. Age of the child, sex of the household head, timely initiation of breastfeeding, current breastfeeding status, number of antenatal care visit, maternal education, and region were significantly associated with inadequate meal frequency. The spatial distribution of inadequate meal frequency was showed significant variation across Ethiopia (Global Moran’s I = 0.164, p-value <0.001). A total of 38 significant clusters were detected through SaTScan analysis, from these the 22 primary clusters were located in Somali and Harari.

**Conclusion and recommendation:**

The prevalence of inadequate meal frequency was high in Ethiopia and had significant clustering patter. Significant hotspot clusters were located in Somali, northern Afar, Harari, Amhara, Gambela, and eastern South nation nationalities and peoples’ region. Therefore, public health interventions which enhance breastfeeding practice, optimal number of antenatal care visits, educational empowerments should target hotspot areas to decrease inadequate meal frequency practice.

## Introduction

According to “Article 27” of United Nations Convention on the Rights of the Child, every infant and child has the right to get adequate nutrition for the reason that good nutrition is essential for children to reach their full potential [1,2]. However, according to World Health Organization (WHO) report nearly 45% of deaths among children are attributed to undernutrition [3]. In the first two years of life, cognitive and physical development occurs at a stunning pace [4]. Therefore, proper infant and young child feeding (IYCF) practices including minimum meal frequency (MMF) are key factors which are important to achieve the child’s nutritional requirements, improve child growth and development, and reduces child morbidity and mortality [5–7].

Without achieving daily MMF, infants and young children are prone to malnourishment (mostly stunting and micronutrient deficiencies) and increased risk of diseases and death [8]. More than two–third of child deaths are associated with inappropriate feeding practices which occur during the first two years of life [5,6,9]. In addition, malnourished children are at higher risks of class absenteeism, early dropouts, low enrollment, and consequently low school performance which is costly to the families and educational systems of countries [5,10,11].

Globally, there are about 149 million stunted and about 45 million wasted under-five children [3,12]. Half of under-five stunted children live in Asia (South Asia: 36%, East Asia and pacific: 14%), and more than one-third live in Africa (Sub-Saharan Africa (SSA) contribute 38%), whereas more than two-thirds of all under-five wasted children live in Asia (South Asia: 55%, East Asia and pacific: 13%) and more than one-quarter live in Africa (SSA contribute 24%) [2,12]. From these evidence we can understood that the SSA region is the highly affected area by undernutrition in the world. In the SSA region, suboptimal IYCF practice, poor quality of complementary foods, and micronutrient deficiencies largely contribute to the high mortality among infants and young children [13]. The International Food Policy and Research Institute indicated that to prevent nutritional deficiencies and to attain nutritional needs, child feeding practice in the first 2 years of age requires special attention in Africa [14].

For optimal child growth and development solid, semi-solid, and soft foods should be introduced starting at six months of age [15,16]. The minimum number of daily meal frequency for infant and young children is a proxy indicator for meeting energy requirements that depends on their breastfeeding status and age. Breastfed children age 6–8 months are considered to be fed with a MMF if they receive solid, semisolid, or soft foods at least twice a day. Breastfed children age 6–23 months are considered to be fed with a MMF if they receive solid, semisolid, or soft foods at least three times a day. Non-breastfed children age 6–23 months are considered to be fed with a MMF if they receive solid, semisolid, soft foods or milk feeds at least four times a day and if at least one of the feeds is a solid, semisolid, or soft food [7,17,18].

Based on previous studies done in different settings, factors were identified to be significantly associated with minimum meal frequency and IYCF practices. These include: age of child [5,6,19–24], sex of the child [24], sex of household head [16,25], media exposure [6,26], maternal working status [16], maternal decision making power on household activities [6], timely initiation of breastfeeding [27], current breastfeeding status [27], parity [5,6], postnatal visit [6,20,23,24], household wealth index [20,22,23,28], maternal age [2], maternal education [2,19,21,29], place of delivery [30], number of antenatal care (ANC) visits [22], place of residence [19,23], and region [2,20] were found associated with minimum meal frequency.

In Ethiopia, malnutrition is a major public health problem among infants and young children. About 37%, 7%, and 21% of under-five children were stunted, wasted and underweight respectively [31]. Efforts are being made in implementing a multi-sectoral nutritional interventions to end the high burden of undernutrition in Ethiopia by 2030 [32,33].

The United Nations Sustainable Development Goal-2 (SDG-2) aims to end all forms of malnutrition by 2030 [34]. Ethiopia has witnessed encouraging progress in reducing malnutrition over the past fifteen years. Stunting, wasting and underweight were declined by 14%, 5% and 12%, respectively from 2005 to 2019. However, malnutrition remains a public health problem that Ethiopia still needs to continue significant investment in nutrition. Currently the Sustainable Undernutrition Reduction in Ethiopia (SURE) and Seqota Declaration (SD) government-led integrated programs are available to reduce undernutrition and improve the infant and young child feeding practices in the country. The Ethiopia national nutrition program targets to decrease stunting and wasting up to 19% and 3% by 2029 respectively through (1) promotion of optimal nutrition for infants and young children, (2) emergency nutrition response and management of acute malnutrition, and (3) multi-sectoral coordination strategies [32].

Even though studies were conducted on the factors associated with meal frequency status among children aged 6–23 months in different settings of Ethiopia [5,6,35], the risky areas (hotspots) of inadequate meal frequency were not identified. In addition, these studies were had limitation on generalizability to the national children aged 6–23 months.

Updated evidence on IYCF practices at a national level will assist the Ethiopia national nutrition program to monitor the changes in the feeding practices and design interventions that are appropriate to increase the recommended feeding practices and thereby plays role in nutrition target achievement. Mothers and caregivers need to be supported by health professionals to initiate and maintain appropriate IYCF practices [8]. This can be done through detecting the geographic variation of inadequate meal frequency which is important to prioritize and design area-targeted intervention programs by policy makers to reduce inadequate meal frequency feeding practice in high-risk areas. Thus, this study was aimed to explore the spatial patterns of inadequate meal frequency feeding practice and to investigate associated factors among children aged 6–23 months in Ethiopia using a national data.

## Methods

### Study design and period

The 2019 mini EDHS data was collected through cross-sectional study design, analysis was done to investigate the spatial patterns of inadequate meal frequency and its associated factors among children aged 6–23 months. The 2019 mini EDHS was the second mini demographic and health survey conducted by the Ethiopian Public Health Institute (EPHI) in collaboration with the Ethiopian Central Statistical Agency (CSA) and the Ministry of Health. The survey was conducted from 21 March 2019 to 28 June 2019 based on a nationally representative sample that provided estimates at the national and regional levels and for urban and rural areas [31].

### Study setting

The study was conducted in Ethiopia, a country located in East Africa at a geographical location of 9°8’42’’ North latitude and 40°29’22.8’’ East longitude [36]. Administratively, Ethiopia is divided into nine regions (Tigray, Afar, Amhara, Oromia, Somalia, Benishangul-Gumuz, Southern Nation Nationality and People’s Region (SNNPR), Gambela, and Harari) and two self-administrative cities (Addis Ababa and Dire Dawa). Each of nine administrative regions and two administrative cities are organized into zones, districts, and kebeles [31].

### Sampling procedure, sample size and participants

**Fig 1:**
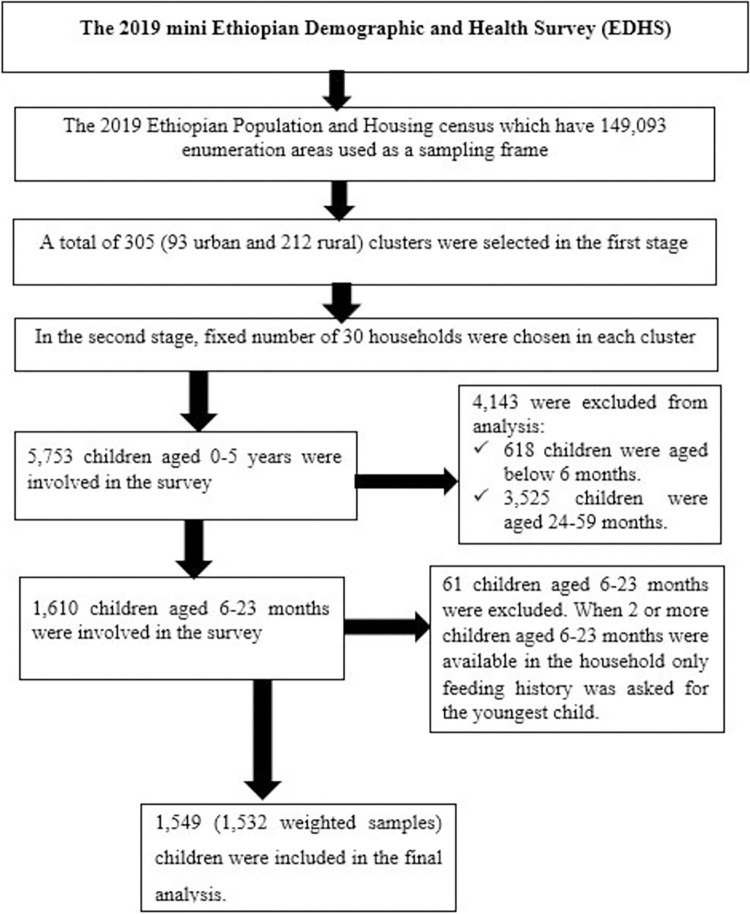
Flow diagram of the 2019 mini EDHS sampling design and data extraction process.

A two-stage stratified cluster sampling technique was used. At the first stage a total of 305 (93 in urban and 212 in rural areas) enumeration areas (EAs) were chosen with probabilities proportionate to EA size based on the 2019 Ethiopian Population and Housing Census (EPHC) frame. In the second stage, fixed number of 30 households in each cluster were chosen with an equal probability systematic selection. For this study we used Kids Record (KR) dataset which contain maternal and child information and variable extraction was performed based on the available literatures. Infant and young children aged 6–23 months during the survey period were included. Additional information on the data collection process, data quality control, sampling, questionnaires used in the survey are explained elsewhere [31]. The data including participant characteristics and coordinate files used for this study were derived from http://www.dhsprogram.com up on an official request and permission. Shape file was obtained from CSA of Ethiopia database. A total weighted sample of 1532 children aged 6–23 months were included in the study (**Fig 1**).

### Variables of the study and measurement

#### Dependent variable

Meal frequency status was the dependent variable in this study. It is the number (frequency) of meals the child receive within 24 hours prior to the interview. The MMF score for children aged 6–23 months is defined as the proportion of breastfed and non-breastfed children 6–23 months of age who received solid, semi-solid or soft foods for the minimum number of times or more during a day before interview. Minimum is defined as 2 times for breastfed infants 6–8 months, 3 times for breastfed children 9–23 months, 4 times for non-breastfed children 6–23 months (including milk feeds). This outcome variable was built based on the mother’s 24-hour recall of meal frequency of the child [18]. If the child achieve the MMF it was coded as “adequate = 0” whereas if the child didn’t achieve the daily MMF it was coded as “inadequate = 1”.

**Independent variables and their category. Individual-level variables:** age of child in months [6–11,12–17,18–23], sex of child (male, female), sex of household head (male, female), timely initiation of breastfeeding (no, yes), current breastfeeding status (no, yes), parity (primipara, multipara, grand-multipara), postnatal visit (no, yes), household wealth index (poorest, poorer, middle, richer, and richest), maternal age [15–49], maternal education(no, primary, secondary, and higher), place of delivery (home, health institution), and number of antenatal care visits (zero, 1–3, and ≥4).

**Community-level variables:** community ANC utilization (low, high), community poverty (low, high), community education (low, high), place of residence (urban, rural) and region (Tigray, Afar, Amhara, Oromia, Somalia, Benishangul-Gumuz, SNNPR, Gambela, Harari, Addis Ababa, and Dire Dawa).

#### Operational definitions

Individual-level variables were operationally defined as follows based on 2019 mini EDHS report [31].Variables were ascertained based on the maternal response for the interview questions.

**Timely initiation of breastfeeding:** the time in which the child put in to breast milk after delivery. It was considered as timely initiated (yes) when the child put in to breast milk within the first hour after birth.

**Current breastfeeding status:** it is the breastfeeding status of the child within 24 hours prior to the interview. It was considered as on breastfeeding (yes) when the child was on breast milk within 24 hours preceding the interview.

**Postnatal visit:** it is postnatal health checkup for the newborn within two months after birth. It was recorded as yes when the child had health checkup within 2 months after birth.

Except place of residence and region, community-level variables were constructed by aggregating from individual-level factors. Community-level variables were categorized into low and high using median value based on their distribution (all aggregated values were skewed). Previous literatures were also use aggregated community variables from individual-level characteristics categorized using mean or median value [16,37]. Place of residence and region were originally recorded, so aggregation wasn’t done.

**Community ANC utilization:** it is an aggregated variable from number of ANC visits, indicating the percentage of women belongs to a community of women who had below four ANC visits. It was considered as low if the percentage of women who had be1ow four ANC visits was lower than 60% (median) in the community whereas it was considered as high if the percentage was at least 60%.

**Community poverty:** it is an aggregated variable from household wealth index, indicating the percentage of children in the community belongs to either poorer or poorest households. It was considered as low if the percentage was lower than 33.3% (median) whereas it was considered as high if the percentage was at least 33.3%.

**Community education:** it is an aggregated variable from women’s educational status, indicating the percentage of women in the community belongs to either secondary or higher education. It was considered as low if the percentage was lower than 17.6% (median) whereas it was considered as high if the percentage was at least 17.6%.

#### Data management and analysis

After the data was obtained from MEASURE DHS website and the KR Stata dataset was used during analysis. To restore the representativeness of the data, weighted samples were used during analysis which helps to compensate for the unequal probability of selection between the strata occurred during data collection process. Stata version 16, ArcGIS version 10.8 and SaTScan version 9.6 software were used for analysis and visualization.

### Multilevel analysis

The mini EDHS data has hierarchical nature and children are nested within a cluster and we expect that children within the same cluster may be more similar to each other than children found in different clusters. This violates observation independency and equality of variance assumptions of the classical logistic regression model. This implies that the need to take into account the between cluster variability by using a mixed-effect model [38]. Therefore, multilevel mixed-effect logistic regression model was fitted to estimate the association between the individual and community-level variables and inadequate meal frequency. Intra-class Correlation Coefficient (ICC), Median Odds Ratio (MOR), and Proportional Change in Variance (PCV) were estimated to measure the variability explained by cluster variation.

Intra-class correlation coefficient (ICC) is used to determine the degree of within-cluster dependence or the degree of heterogeneity across clusters [39,40]. In this study ICC was used to quantify the amount of individual variability on inadequate meal frequency attributed to cluster variation [40,41].

$$ICC=\frac{\sigma^{2}}{\sigma^{2}+\frac{\pi^{2}}{3}}$$ (1)

Where; *σ*^2^ is the cluster level variance and $\frac{\pi^{2}}{3}$ is the individual-level variance.

The MOR is the median value of the odds ratios between the area at highest risk and the area at lowest risk when we pick two random clusters. It can be interpreted as the increased amount of risk that happen when someone move from low risk cluster to a higher risk cluster. In this study, the MOR shows the individual increased risk of inadequate meal frequency when someone moves from cluster with low probability of inadequate meal frequency to area with high risk of inadequate meal frequency. If MOR is equal to one, there would be no differences between clusters in the probability of inadequate meal frequency [40].

$$MOR={exp}^{(\sqrt{2*\sigma^{2}*0.6745)}}=\exp\left(0.95\sqrt{\sigma^{2}}\right)$$ (2)

Where; *σ*^2^ is the cluster level variance, and 0.6745 is the 75^th^ centile of the cumulative distribution function of the normal distribution with mean of 0 and variance of 1.

Proportional (percentage) change in variance (PCV) is a measure of total variation attributed to individual-level factors and community-level factors in the multilevel model as compared to the null model [40].

$$PCV=\frac{\sigma^{2}null-\sigma^{2}var}{\sigma^{2}null}$$ (3)

Where; *σ*^2^*null* is variance of the null model, and *σ*^2^*var* is variance of the model with individual-level or community-level variables.

Four models were constructed for multilevel mixed-effect logistic regression analysis. The first model was an empty model without explanatory variables to determine the extent of cluster variation on inadequate meal frequency; the second model was constructed with individual-level variables and inadequate meal frequency; the third model was constructed with community-level variables and inadequate meal frequency whereas the fourth model was constructed with individual and community variables simultaneously and inadequate meal frequency.

Deviance and Log-Likelihood (LL) were used to compare and select the best-fitted model and a model with the lowest deviance and largest LL was considered the best-fitted model (Model-Ⅳ). Multicollinearity was checked using the variance inflation factor (VIF) which indicates the absence of multi-collinearity since all variables were had VIF < 2 and tolerance greater than 0.4. The final model (a model with individual and community-level variables) was chosen since it had lowest deviance and largest LL values. Finally, Adjusted Odds Ratio (AOR) with its 95% confidence interval (CI) was reported, and variables with p-value < 0.05 in the multivariable multilevel mixed-effect logistic regression analysis were considered as significant predictors of inadequate meal frequency.

**Spatial analysis of inadequate meal frequency. Spatial autocorrelation analysis:** The spatial distribution of inadequate meal frequency among children aged 6–23 months was visualized through spatial projection. Each dots represent one cluster and the color indicates its corresponding prevalence of inadequate meal frequency in the cluster. The global spatial autocorrelation (Global Moran’s I) statistics was used to determine whether the distribution of inadequate meal frequency among infants and young children in Ethiopia was dispersed, clustered or randomly distributed. Global Moran’s I statistic is used to measure spatial autocorrelation by taking the entire dataset and produce a single output value that ranges from -1 to +1. Moran’s I value close to −1 indicates dispersed pattern, whereas Moran’s I close to +1 indicates clustered pattern and Moran’s I value of 0 reveals complete randomness. A statistically significant Moran’s I (p < 0.05) value indicates the presence of non-random distribution [42]. Moran’s I value is based on Tobler’s first law of geography which states “everything is related to everything else, but close things are more related than distant things”[43]. Based on this law, inadequate meal frequency prevalence should be more similar among neighboring clusters than among distant clusters. We used inverse-distance conceptualization for spatial relationships (Tobler’s first law of geography) and Euclidean distance (straight line distance between two points) method through raw standardization (to minimize biased distribution of spatial features) to estimate the global spatial autocorrelation. Global Moran’s I is a global spatial autocorrelation statistics that used to determine the overall degree of spatial autocorrelation in the dataset. However, the degree of spatial autocorrelation might significantly vary across space. Hence, local indicator of spatial autocorrelation (LISA) statistics can provide disaggregated estimates at different place which allows to visualize the spatial autocorrelation variation across different areas [42]. We used LISA to detect local spatial autocorrelation of inadequate meal frequency. Dots with green color indicate low proportion of inadequate meal frequency whereas red color dots indicate high proportion of inadequate meal frequency.

**Hotspot analysis:** The Getis-Ord Gi* statistic was used to provide additional information on LISA indicating the intensity of core hotspot (high prevalence of inadequate meal frequency) or coldspot (low prevalence of inadequate meal frequency) clusters [44]. This method helps to evaluate the strength of clustering across areas.

**SaTScan analysis:** Spatial scan (SaTScan) statistical analysis was conducted to identify significant primary and secondary clusters of inadequate meal frequency based on Bernoulli distribution. This analysis uses a circular scanning window that goes across areas of the study. Children aged 6–23 months who had inadequate meal frequency were considered as cases while those who had adequate meal frequency were considered as controls to fit the Bernoulli model. The most likely performing cluster was the scanning window with a maximum likelihood. The distribution and statistical significant clusters were explored by Monte Carlo replication of data with greater than 999 replications to ensure adequate power in cluster identification process [45,46].

**Spatial interpolation analysis:** In order to produce smooth surfaces of inadequate meal frequency prevalence for visualization and data generation across Ethiopia, the possible value of un-sampled areas can be estimated through interpolation technique [44]. Empirical Bayesian Kriging interpolation technique was used to predict the prevalence of inadequate meal frequency in Ethiopia. It is a robust method of spatial interpolation which account error introduced by estimating the semivariogram [47].

**Spatial regression analysis:** Spatial regression models are important to explore the relationship between distribution of a certain conditions and other different socio-demographic, environmental, economic characteristics of the population across places [48].

The Ordinary Least Square (OLS) regression and Geographic Weighted Regression (GWR) spatial models were used to explore spatial relationships between inadequate meal frequency and independent variables. The OLS regression is a global model with a single equation to estimate the relationship between the outcome and independent variables with the assumption of spatially consistent relationships. Hence, we aimed to visualize the spatial relationship between prevalence of inadequate meal frequency calculated at each cluster and other eight explanatory variables from the multilevel mixed-effect analysis based on their statistical significance.

OLS model diagnostic parameters which are; adjusted-R^2^, Jarque-Bera statistic, Joint F and Wald statistic, and Koenker (BP) statistic were checked. Adjusted-R^2^ measures the amount of inadequate meal frequency variation explained by the model (higher values indicate better predictive performance). The Jarque-Bera statistic indicates whether the model predictions are biased or not. Insignificant Jarque-Bera statistic indicates unbiased model. The overall model significance was evaluated by Joint F and Wald statistics. Koenker statistic is a measure of the relationship stationarity in the model if it is statistically insignificant whereas significant value indicates non-stationary relationship between independent and outcome variables. Multicollinearity was assessed using the Variance Inflation Factor (VIF). Based on Koenker (BP) statistic, there was non-consistent spatial relationship between inadequate meal frequency and independent variables (p-value < 0.001). Residuals spatial autocorrelation statistic was estimated, indicating that the OLS model was biased due to non-normal distribution of residuals [49,50].

Since the OLS model assumptions were not fulfilled due to heterogeneous spatial relationship as indicated by both significant Koenker statistics and clustering residual distribution, so GWR model was considered to handle it. Similar independent variables were considered as OLS model. Finally the two models were compared using AICc and adjusted-R^2^ values. A model with higher value of adjusted-R^2^ and lower value AICc was the best-fit model (GWR). Based on the best-fit model, the coefficients of significant spatial predictors were visualized.

### Missing data management

Missing data was handled according to the DHS guideline. Thus, all analysis was based on complete observations.

### Ethical consideration

Permission for data access and ethical approval was obtained from MEASURE DHS through the online formal request at www.dhsprogram.com. All methods were carried out in accordance with relevant guidelines of the Demographic and Health Surveys (DHS) program. Publicly available data with no personal identifier were used. Participants and the general public were not involved in the design and analysis phase of this investigation.

## Results

### Characteristics of study participants

A total weighted samples of 1532 children aged 6–23 months were included in the study. Out of these more than half (52.61%) of children were males. Nearly nine children out of ten (86.23%) were from male headed households. Majority of children, 81.98% and 81.59% were initiate breastfeeding within the first hour after birth and on breastfeeding during the survey respectively. More than half (54.44%) of children were delivered in health institutions. One-fourth (25.07%) of the mothers were didn’t have history of antenatal care visits during their pregnancy period of the youngest child. Regarding postnatal checkup within two months after birth, 86.62% of children didn’t have postnatal checkup. Around 45% of mothers hadn’t formal educational and 20.37% child live in poorest households. Community women’s low levels of less than four ANC utilization, at least secondary education and poverty were 47.28%, 65.15% and 45.12%, respectively. Majority (72.13%) of study participants were reside in rural areas and 37.6% of children were from Oromia region (**Table 1**).

**Table 1:** Individual and community-level characteristics of children and their mother in Ethiopia, mini EDHS 2019.

Characteristics	Category	Weighted frequency (n = 1532)	Weighted percentage
Age of child	6–11	502	32.77
	12–17	565	36.88
	18–23	465	30.35
Sex of child	Male	806	52.61
	Female	726	47.39
Sex of household head	Male	1321	86.23
	Female	211	13.77
Timely initiation of breastfeeding	No	276	18.02
	Yes	1256	81.98
Current breastfeeding status	No	282	18.41
	Yes	1250	81.59
Postnatal check within two months	No	1327	86.62
	Yes	205	13.38
Place of delivery	Home	698	45.56
	Institution	834	54.44
Number of antenatal visits	Zero-visit	384	25.07
	1–3	463	30.22
	≥4	685	44.71
Parity	Primipara (1)	376	24.54
	Multipara (2–4)	710	46.35
	Grand-multipara (≥5)	446	29.11
Maternal age	15–19	118	7.70
	20–24	382	24.94
	25–29	493	32.18
	30–34	262	17.10
	35–39	189	12.33
	40–44	75	4.90
	45–49	13	0.85
Maternal educational status	No-education	691	45.10
	Primary	631	41.19
	Secondary	125	8.16
	Higher	85	5.55
Household wealth index	Poorest	312	20.37
	Poorer	325	21.21
	Middle	285	18.60
	Richer	273	17.82
	Richest	337	22.00
Community ANC utilization	Low	724	47.28
	High	808	52.72
Community education	Low	998	65.15
	High	534	34.85
Community poverty	Low	691	45.12
	High	841	54.88
Residence	Urban	427	27.87
	Rural	1105	72.13
Region	Tigray	108	7.05
	Afar	21	1.37
	Amhara	339	22.13
	Oromia	576	37.60
	Somali	95	6.20
	Benishangul	17	1.11
	SNNPR	304	19.84
	Gambela	7	0.46
	Harari	4	0.26
	Addis Ababa	51	3.33
	Dire Dawa	10	0.65

### Prevalence of inadequate meal frequency

**Fig 2:**
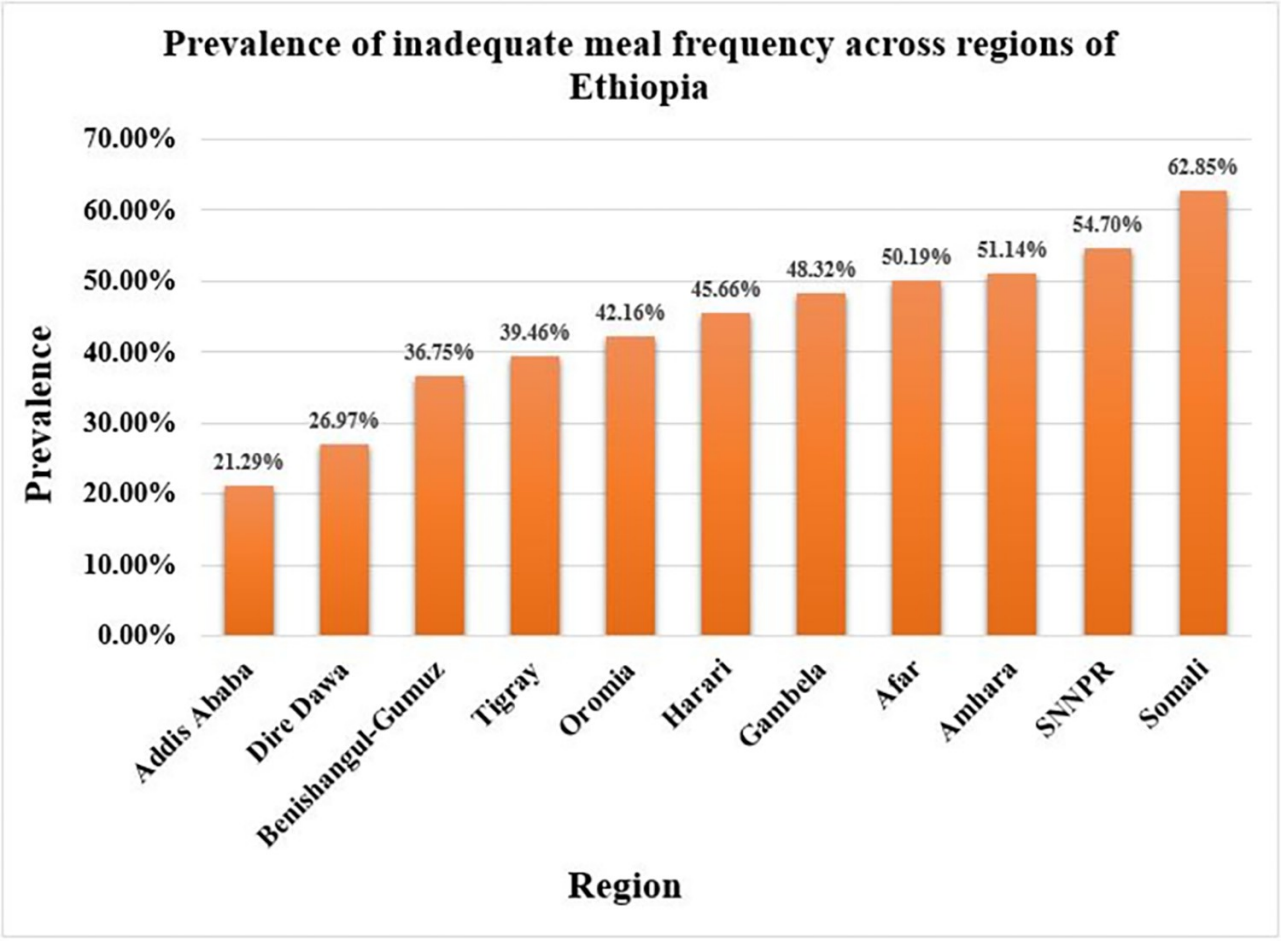
Prevalence of inadequate meal frequency among children aged 6–23 months in Ethiopia, mini EDHS 2019.

The prevalence of inadequate meal frequency among children aged 6–23 months was 47.03% (95% CI: 44.54%, 49.53%) in Ethiopia. The lowest and highest prevalence of inadequate meal frequency was observed in Addis Ababa (21.29%) and Somali (62.85%), respectively (**Fig 2**).

### Factors associated with inadequate meal frequency

#### Random effect analysis

The amount of variability on inadequate meal frequency among children aged 6–23 months explained by cluster variation was 13.53% (95% CI: 8.64, 20.56) based on the estimated ICC whereas 86.47% of variability on inadequate meal frequency was explained by individual-level variations. When a child moves from area with low risk to high risk of inadequate meal frequency, 98% increased risk of having inadequate meal frequency was observed (MOR = 1.98; 95% CI: 1.64, 2.32). The amount of variability explained by individual-level factors, community-level factors, and both individual and community-level factors together was 36.19%, 62.97% and 58.59% respectively. According to the Likelihood Ratio (LR) test (LR test mixed-effect vs. logistic model, p-value < 0.001), models which considered clustering effect (mixed-effect) were found better-fitted the data than the classical logistic regression model. A model with individual and community-level factors (Model-Ⅳ) was found the best-fit model since it had smallest deviance and largest LL (**Table 2**). Therefore, based on the best-fit model factors associated with inadequate meal frequency were reported.

**Table 2:** Random effect analysis results and model comparison.

Random effect	Model-Ⅰ (empty)	Model-Ⅱ	Model-Ⅲ	Model-Ⅳ
ICC %	13.53 (8.64, 20.56)	8.63 (4.38, 16.29)	5.01 (1.89, 12.61)	5.60 (2.14, 13.89)
MOR	1.98 (1.64, 2.32)	1.70 (1.37, 2.03)	1.49 (1.19, 1.79)	1.52 (1.20, 1.85)
PCV %	Reference	36.19	62.97	58.59
**Model fitness**				
LL	-1049.10	-965.22	-1005.92	-951.19
Deviance	2098.20	1930.44	2011.84	1902.38

### Fixed effect analysis

A total of seventeen independent variables were considered in the bi-variable model. From these 16 variables which had a p-value < 0.25 (except sex of the child; p-value = 0.618) were exported to the multivariable multilevel logistic regression model. At 95% confidence level significant factors associated with inadequate meal frequency from the best-fit mode (Model-Ⅳ) were: child age, sex of household head, timely initiation of breastfeeding, current breastfeeding status, number of ANC visit, maternal education, and region.

Children aged 6–11 months were 2.29 times (AOR = 2.29, 95% CI: 1.69, 3.11) more likely to have inadequate meal frequency as compared to children aged 18–23 months. The odds of inadequate meal frequency was increased by 75% (AOR = 1.75, 95% CI: 1.30, 2.35) among children aged 12–17 months as compared to children aged 18–23 months.

The odds of inadequate meal frequency was increased by 35% (AOR = 1.37, 95% CI: 1.02, 1.85) among children who live in male headed households as compared to those children who live in female headed households.

Children who experienced timely initiation of breastfeeding and currently on breastfeeding were had 1.41 (AOR = 1.41, 95% CI: 1.06, 1.87) and 1.86 times (AOR = 1.86, 95% CI: 1.38, 2.50) higher odds of having inadequate meal frequency, respectively.

The odds of inadequate meal frequency was 1.54 (AOR = 1.54, 95% CI: 1.08, 2.21) and 1.35 times (AOR = 1.35, 95% CI: 1.01, 1.81) higher among children born from mothers who had zero and one to three (1–3 inclusive) ANC visits, respectively as compared to children born from mothers who had ≥ 4 ANC visits.

Children born from mothers who hadn’t formal education were had 2.00 times (AOR = 2.00, 95% CI: 1.09, 3.70) higher odds of inadequate meal frequency as compared to children born from mothers who achieve higher education.

The odds of inadequate meal frequency was 2.49 (AOR = 2.49, 95% CI: 1.19, 5.21), 2.44 (AOR = 2.44, 95% CI: 1.16, 5.13), and 2.38 times (AOR = 2.38, 95% CI: 1.08, 5.20) higher among children who reside in Amhara, SNNPR and Gambela, respectively as compared to children residing in Addis Ababa (**Table 3**).

**Table 3:** Bi-variable and multivariable multilevel logistic regression analysis to identify factors associated with inadequate meal frequency among children aged 6–23 months in Ethiopia, mini EDHS 2019.

Characteristics	Category	AOR (95% CI)			
		Model-Ⅰ (empty)	Model-Ⅱ	Model-Ⅲ	Model-Ⅳ
Age of child	6–11		2.30 (1.69, 3.12)***		2.29 (1.69, 3.11)***
	12–17		1.75 (1.30, 2.35)***		1.75 (1.30, 2.35)***
	18–23		1		1
Sex of household head	Male		1.35 (1.01, 1.81)*		1.37 (1.02, 1.85)*
	Female		1		1
Timely initiation of breastfeeding	No		1.43 (1.07, 1.90)*		1.41 (1.06, 1.87)*
	Yes		1		1
Current breastfeeding status	No		1.76 (1.31, 2.36)***		1.86 (1.38, 2.50)***
	Yes		1		1
Postnatal check within two months	No		1.18 (0.83, 1.68)		1.20 (0.84, 1.70)
	Yes		1		1
Place of delivery	Home		0.98 (0.73, 1.32)		0.96 (0.71, 1.29)
	Institution		1		1
Number of antenatal visits	Zero-visit		1.83 (1.29, 2.58)**		1.54 (1.08, 2.21)*
	1–3		1.55 (1.17, 2.04)**		1.35 (1.01, 1.81)*
	≥4		1		1
Parity	Primipara (1)		1		1
	Multipara (2–4)		0.89 (0.64, 1.23)		0.86 (0.63, 1.19)
	Grand-multipara (≥5)		1.28 (0.86, 1.90)		1.24 (0.83, 1.86)
Maternal age	15–19		1.89 (0.57, 6.26)		1.94 (0.59, 6.34)
	20–24		1.84 (0.59, 5.72)		1.94 (0.63, 5.95)
	25–29		2.20 (0.73, 6.63)		2.27 (0.76, 6.76)
	30–34		1.73 (0.58, 5.19)		1.73 (0.58, 5.13)
	35–39		1.92 (0.63, 5.86)		1.91 (0.64, 5.77)
	40–44		0.84 (0.25, 2.81)		0.84 (0.26, 2.79)
	45–49		1		1
Maternal educational status	No-education		2.08 (1.15, 3.76)*		2.00 (1.09, 3.70)*
	Primary		1.54 (0.87, 2.72)		1.39 (0.78, 2.49)
	Secondary		1.40 (0.74, 2.66)		1.38 (0.73, 2.62)
	Higher		1		1
Household wealth index	Poorest		2.06 (1.36, 3.12)**		1.75 (0.96, 3.20)
	Poorer		1.46 (0.95, 2.22)		1.13 (0.63, 2.03)
	Middle		1.31 (0.86, 1.99)		1.02 (0.59, 1.78)
	Richer		1.01 (0.66, 1.53)		0.78 (0.46, 1.30)
	Richest		1		1
Community ANC utilization	Low			1	1
	High			1.65 (1.22, 2.22)**	1.34 (0.96, 1.87)
Community education	Low			1.44 (1.06, 1.96)*	1.20 (0.84, 1.72)
	High			1	1
Community poverty	Low			1	1
	High			1.35 (0.99, 1.85)	1.01 (0.68, 1.47)
Residence	Urban			1	1
	Rural			0.93 (0.65, 1.34)	0.79 (0.51, 1.22)
Region	Tigray			1.51 (0.74, 3.10)	1.92 (0.90, 4.11)
	Afar			1.72 (0.85, 3.48)	1.33 (0.63, 2.84)
	Amhara			2.20 (1.11, 4.39)*	2.49 (1.19, 5.21)*
	Oromia			1.55 (0.78, 3.06)	1.87 (0.90, 3.90)
	Somali			2.72 (1.31, 5.66)**	1.74 (0.78, 3.86)
	Benishangul			1.33 (0.65, 2.72)	1.37 (0.63, 2.95)
	SNNPR			2.23 (1.12, 4.43)*	2.44 (1.16, 5.13)*
	Gambela			1.91 (0.92, 3.94)	2.38 (1.08, 5.20)*
	Harari			1.95 (0.98, 3.91)	2.06 (0.99, 4.27)
	Addis Ababa			1	1
	Dire Dawa			1.01 (0.50, 2.05)	0.84 (0.40, 1.77)

Notes

*p<0.05

**p<0.01

***p<0.001.

### Spatial analysis results

**Fig 3:**
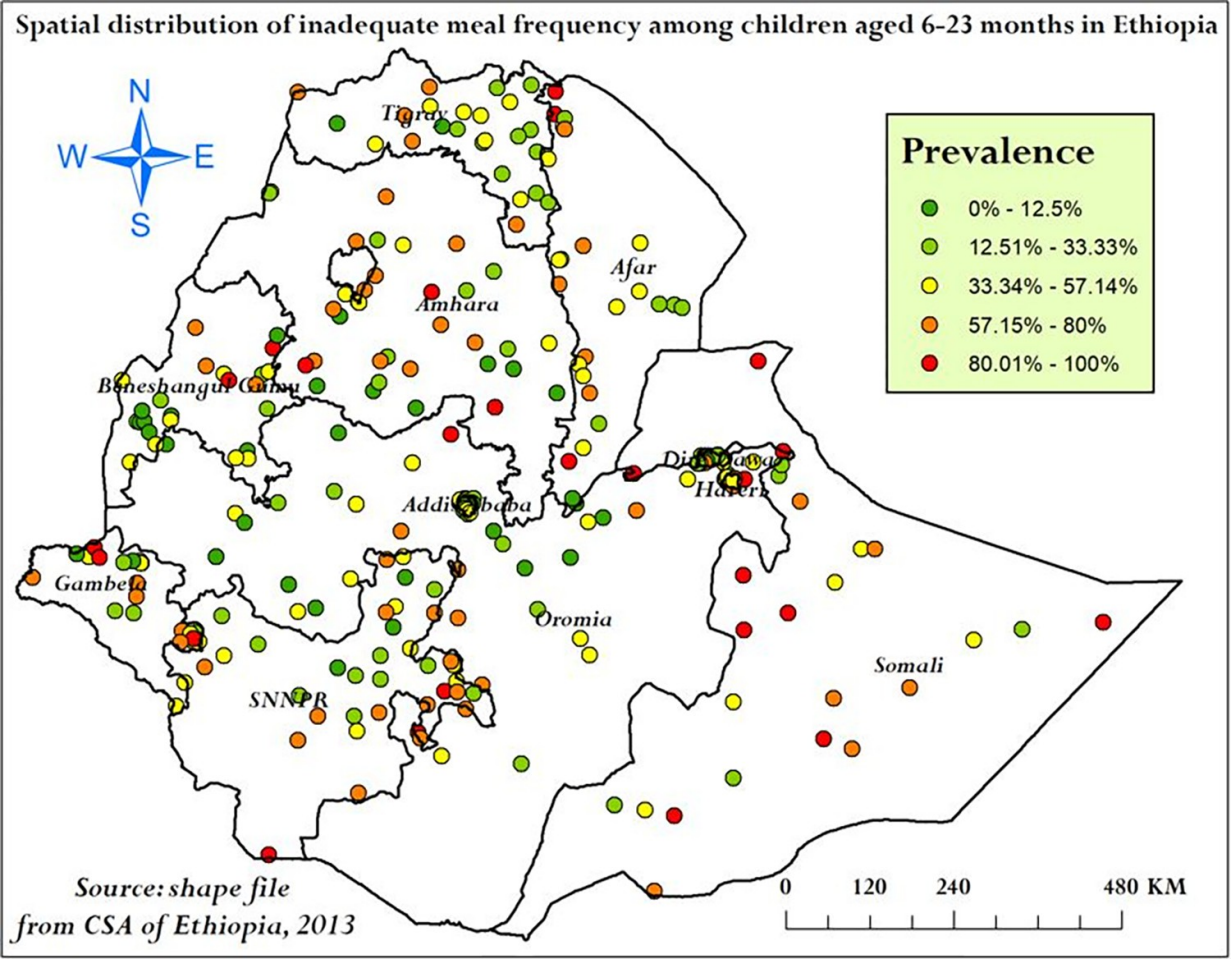
Spatial distribution of inadequate meal frequency among children aged 6–23 months in Ethiopia, mini EDHS 2019.

In the spatial projection analysis, high prevalence of inadequate meal frequency was represented by red colors and low prevalence of inadequate meal frequency was represented by green colors. The prevalence of inadequate meal frequency for each cluster was estimated. Hence, the prevalence of inadequate meal frequency was ranges from 57–100% in the clusters represented by red colors. Majority of these clusters were located in Somali, Amhara, Afar, SNNPR and Gambela regions (**Fig 3**).

**Fig 4:**
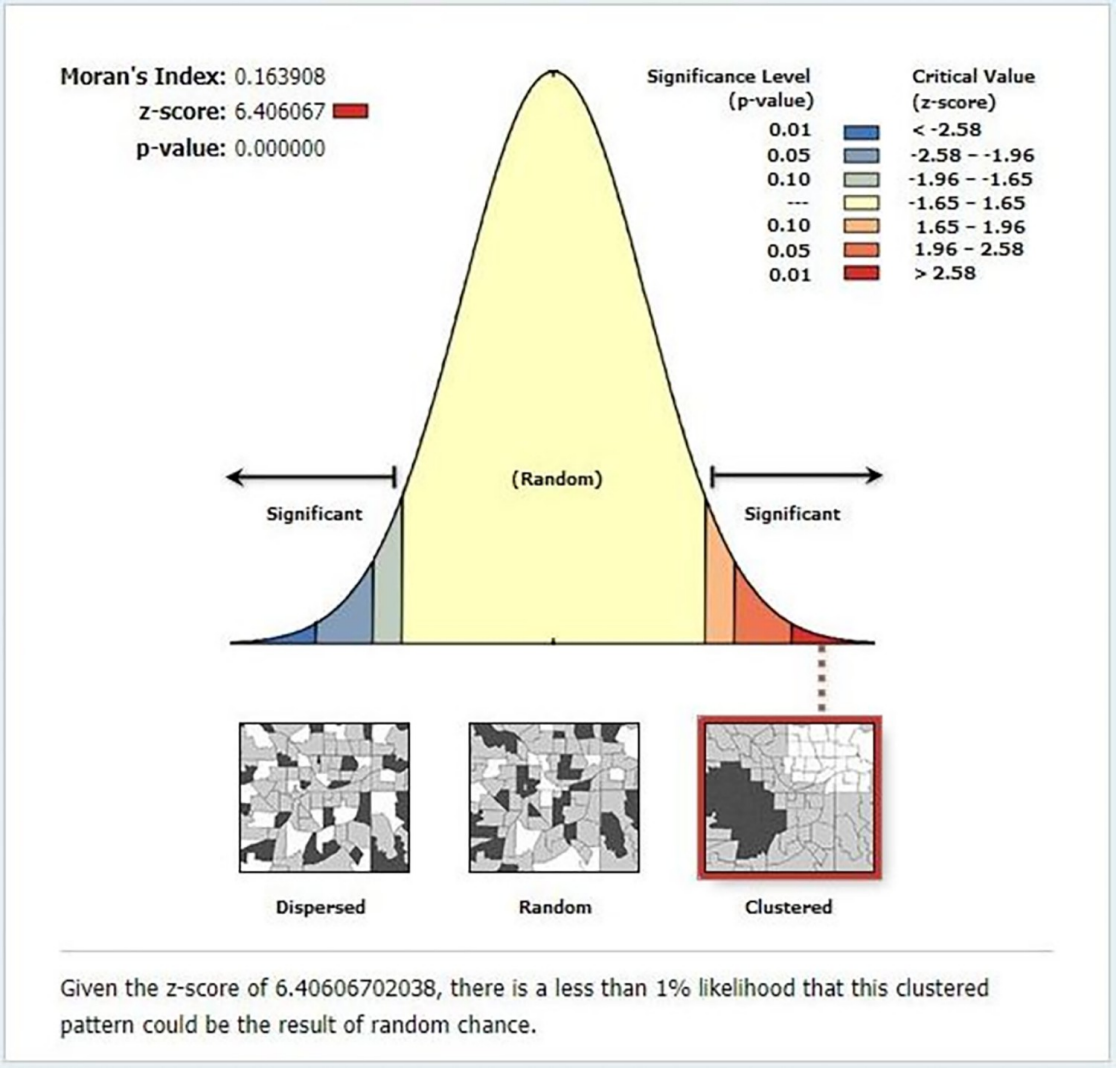
Global spatial autocorrelation analysis of inadequate meal frequency among children aged 6–23 months in Ethiopia, mini EDHS 2019.

**Spatial autocorrelation analysis result:** based on the global spatial autocorrelation analysis, the distribution of inadequate meal frequency was found clustered (Moran’s I = 0.164, Z-score = 6.406, p-value < 0.001) in Ethiopia (**Fig 4**).

**Fig 5:**
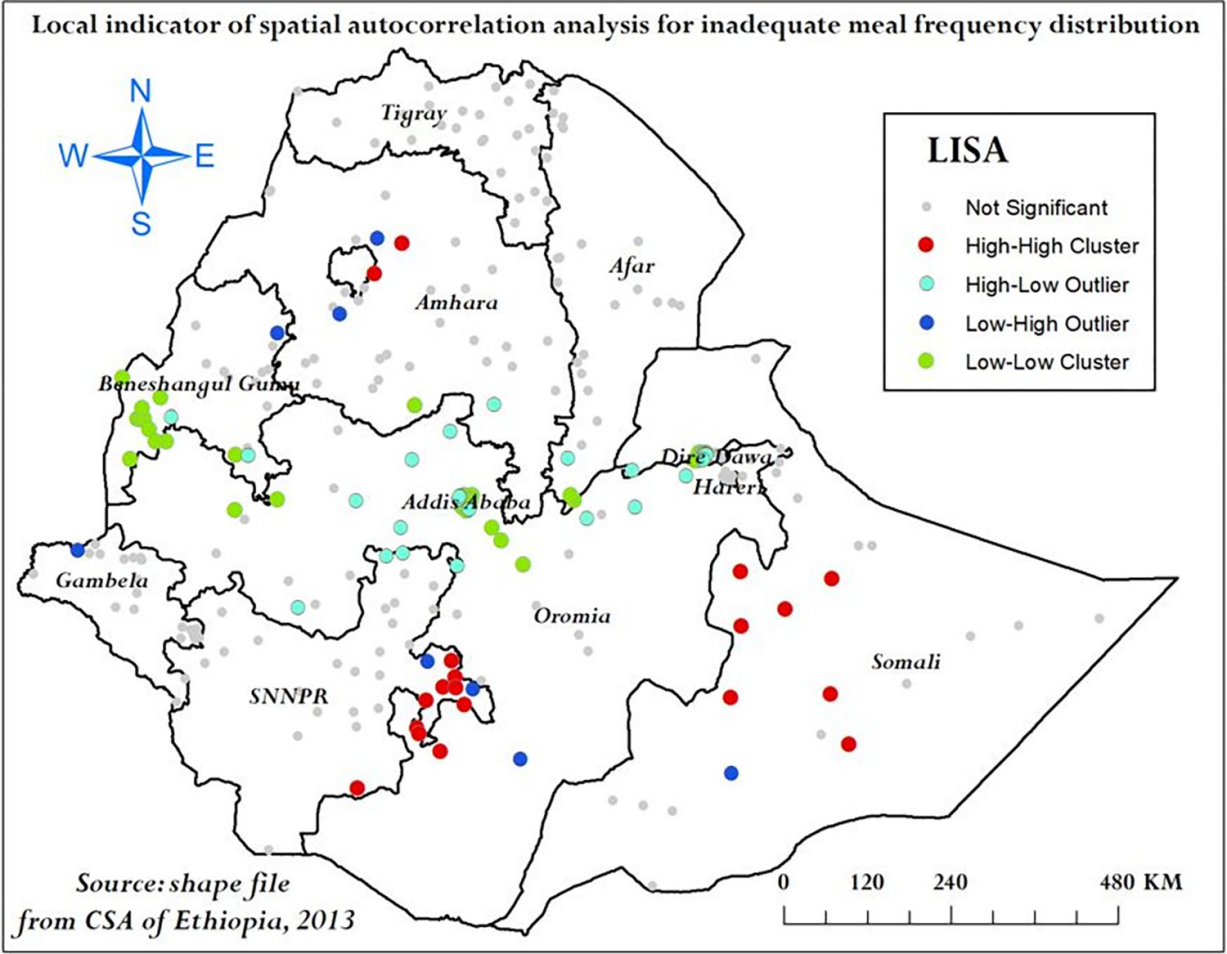
Local indicator spatial autocorrelation analysis of inadequate meal frequency among children aged 6–23 months in Ethiopia, mini EDHS 2019.

The LISA analysis detected local significant clustering patterns of high proportion of inadequate meal frequency in eastern SNNPR, Somali, and northern Amhara. Local significant clustering patterns of low proportion of inadequate meal frequency were detected in southwestern part of Benishangul-Gumuz, Addis Ababa, Dire Dawa, and northern Oromia (**Fig 5**).

**Fig 6:**
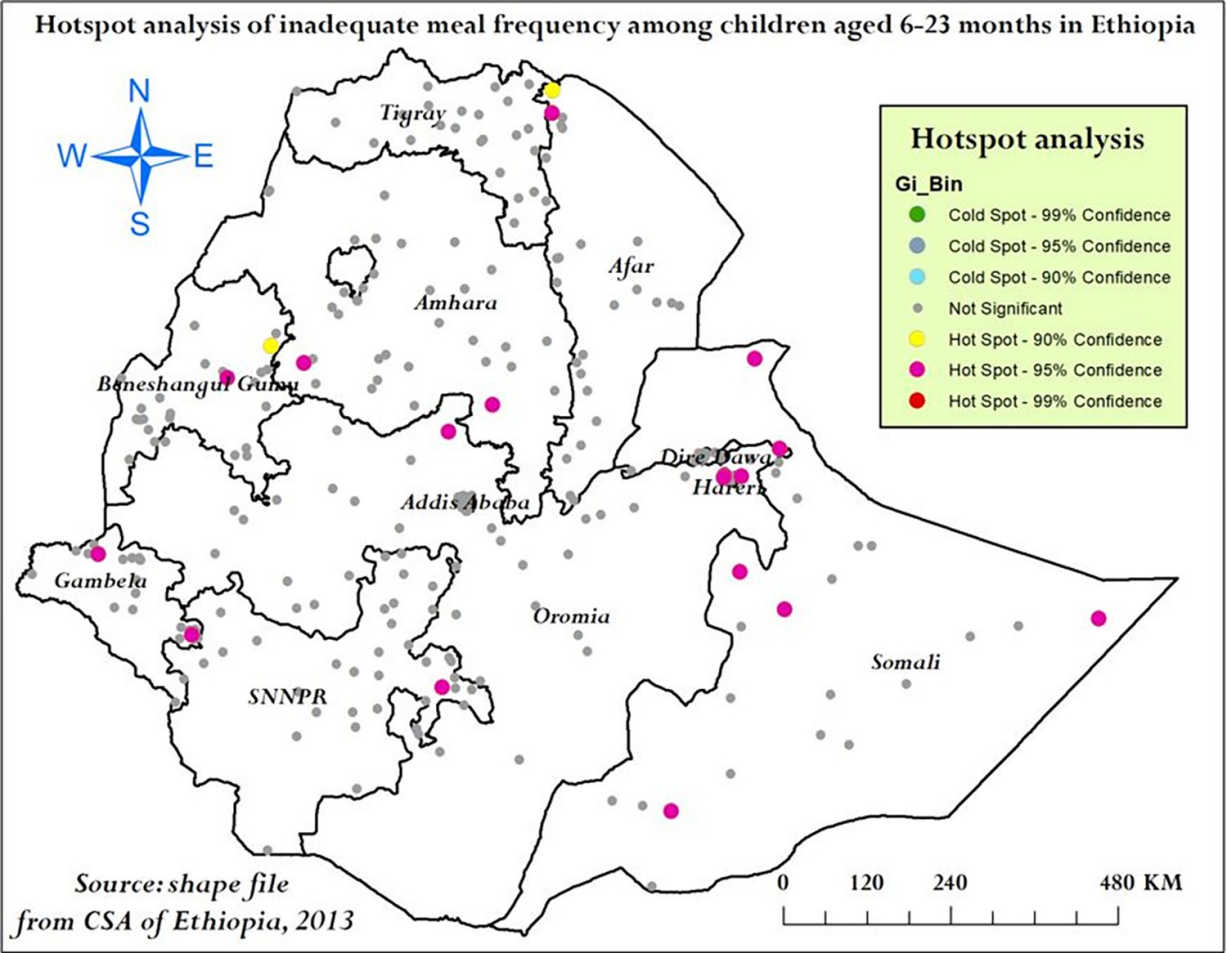
Hotspot analysis of inadequate meal frequency among children aged 6–23 months in Ethiopia, mini EDHS 2019.

**Hotspot analysis result:** In order to detect the intensity of local clustering of inadequate meal frequency, Getis-Ord Gi* analysis was conducted. Based on Getis-Ord Gi* statistics, hotspot areas (high proportion of inadequate meal frequency) were detected at 95% and 90% confidence level. Somali, northern Afar, Harari, Amhara, Gambela and eastern SNNPR were significant hotspot areas of inadequate meal frequency (**Fig 6**).

**Fig 7:**
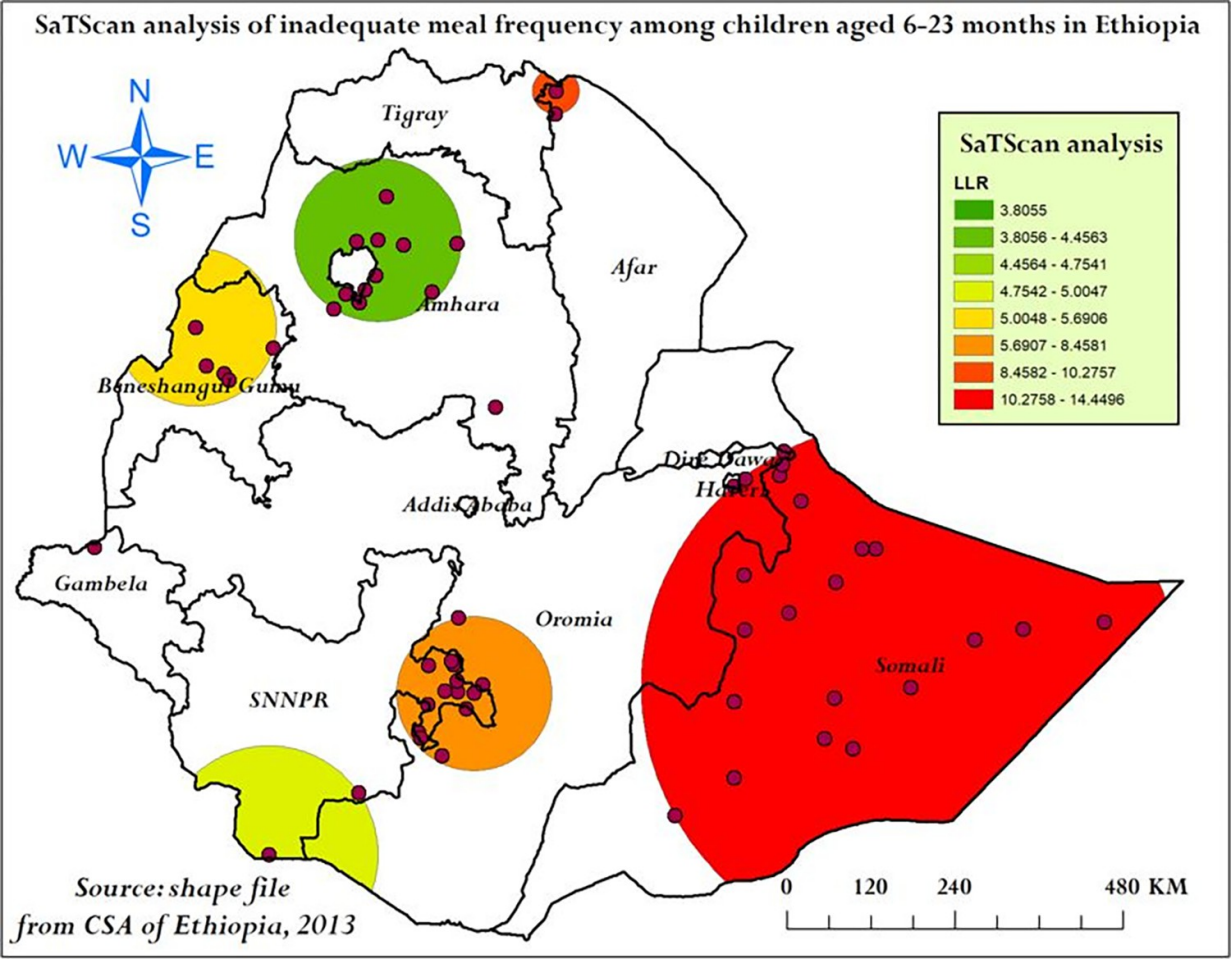
SaTScan analysis of inadequate meal frequency among children aged 6–23 months in Ethiopia, mini EDHS 2019.

SaTScan analysis result: The SaTScan analysis identified a total of 38 significant clusters (within three spatial windows) from the imported 297 clusters, of these 22 clusters were primary (most likely) clusters of inadequate meal frequency located in Somali and Harari centerd at 6.639662 N, 44.465855 E with a radius of 381.83 km, Relative Risk (RR) of 1.57 and Log-Likelihood Ratio (LLR) of 14.45 at p-value < 0.001 (**Table 4)**. This showed that children within the primary spatial window had 1.57 times higher risk of inadequate meal frequency than children located outside the primary spatial window. Two significant secondary clusters were identified by the SaTScan analysis. The first secondary clusters were located in northern Afar and the second secondary clusters were located in eastern SNNPR (**Fig 7**).

**Table 4:** SaTScan analysis of inadequate meal frequency among children aged 6–23 months in Ethiopia, mini EDHS 2019.

Cluster type	Coordinates/radius	N	Cluster locations	Observed cases	Expected cases	RR	LLR	p-value
Most likely clusters	(6.639662N,44.465855E)/ 381.83 km	22	135, 123, 140, 137, 138, 124, 131, 145, 132, 122, 134, 136, 142, 133, 139, 129, 121, 130, 107, 250, 141, 128	87	58.09	1.57	14.450	< 0.001
First Secondary clusters	(14.300432N,39.911831E)/ 32.54 km	2	39, 35	17	8.17	2.11	10.276	0.013
2^nd^ secondary clusters	(6.562485N,38.864491E)/ 109.93 km	14	183, 117, 186, 113, 181, 182, 185, 187, 115, 184, 172, 188, 89, 116	58	39.48	1.51	8.458	0.036
3^rd^ Secondary clusters	(11.267438N,35.292873E)/ 112.65 km	5	159, 160, 158, 161, 162	22	13.16	1.69	5.691	0.368
4^th^ Secondary clusters	(4.495034N,36.230625E)/ 155.19 km	2	193, 202	14	7.72	1.83	5.005	0.620
5^th^ Secondary cluster	(10.236394N,39.142029E)/ 0 km	1	67	6	2.72	2.21	4.754	0.773
6^th^ Secondary clusters	(12.387114N,37.630096E)/ 116.41 km	12	82, 84, 83, 57, 56, 59, 74, 54, 81, 58, 78, 75	45	32.68	1.40	4.456	0.838
7^th^ Secondary cluster	(8.436117N, 33.991501 E) / 0 km	1	218	8	4.08	1.97	3.806	0.962

Note: N; Number of clusters, RR; Relative Risk, LLR; Log-Likelihood Ratio.

**Fig 8:**
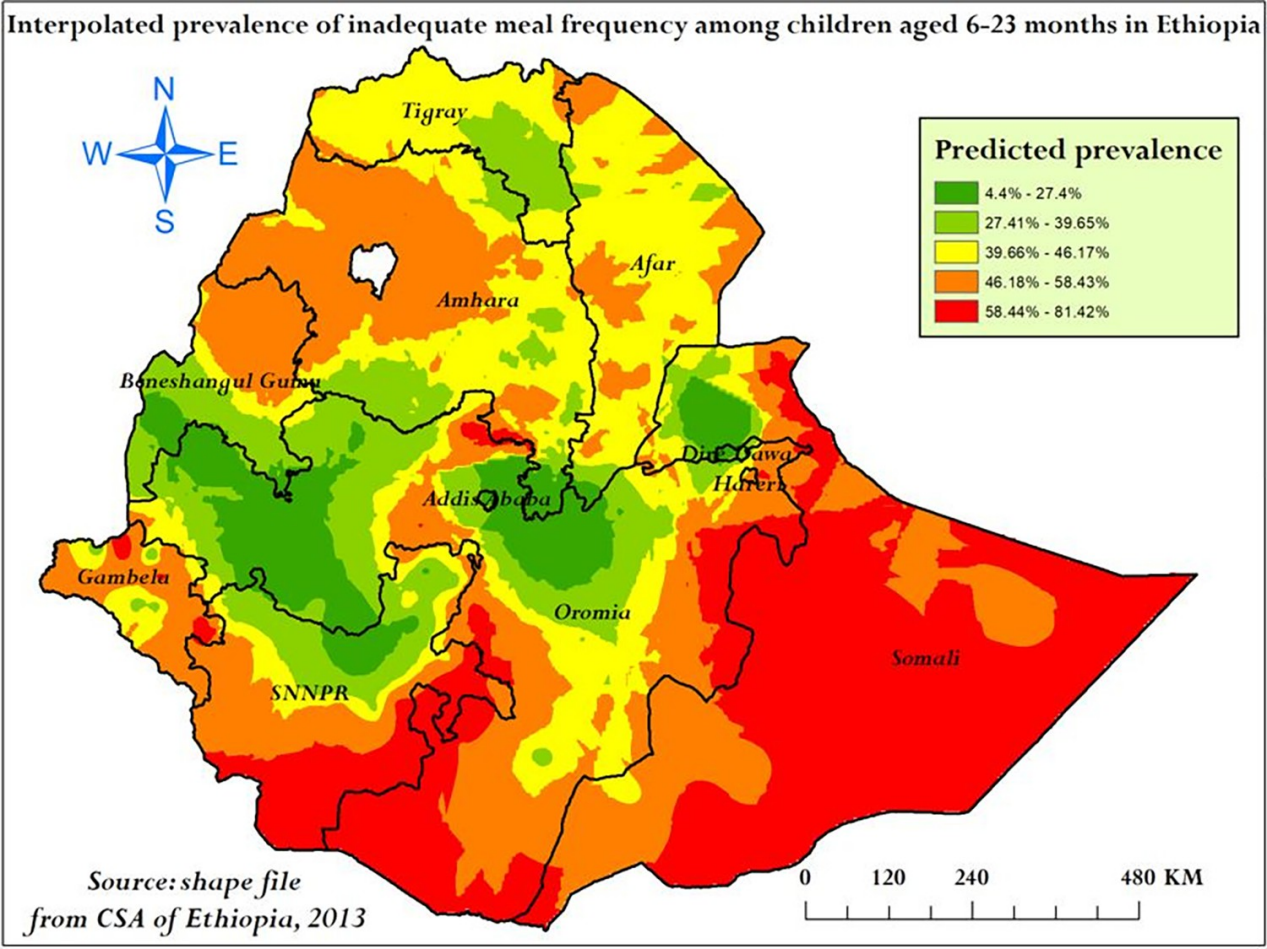
Empirical Bayesian Kriging interpolation analysis of inadequate meal frequency among children aged 6–23 months in Ethiopia, mini EDHS 2019.

**Spatial interpolation analysis:** For the spatial prediction of inadequate meal frequency prevalence across Ethiopia, Empirical Bayesian Kriging interpolation analysis was conducted. Most part of Somali, southern and southeastern SNNPR, and southern Oromia were had 58.44–81.42% predicted prevalence of inadequate meal frequency (**Fig 8**).

**Ordinary least square regression results:** In the OLS analysis, the joint F- and Wald-statistics parameters were significant with p-value <0.001 indicating that the model was statistically significant (it had at least one significant spatial predictor for the variation of inadequate meal frequency). This model explains 19.9% of inadequate meal frequency spatial variability (adjusted-R^2^ = 0.2). Multicollinearity wasn’t an issue during analysis as indicated by VIF reports (< 7.5). According to the Jarque-Bera statistic, residuals weren’t normally distributed (p-value < 0.001), indicating that the OLS model was biased. Koenker statistic was statistically significant (p-value < 0.001), indicating the occurrence of heterogeneous relationship between independent variables and inadequate meal frequency.

**Fig 9:**
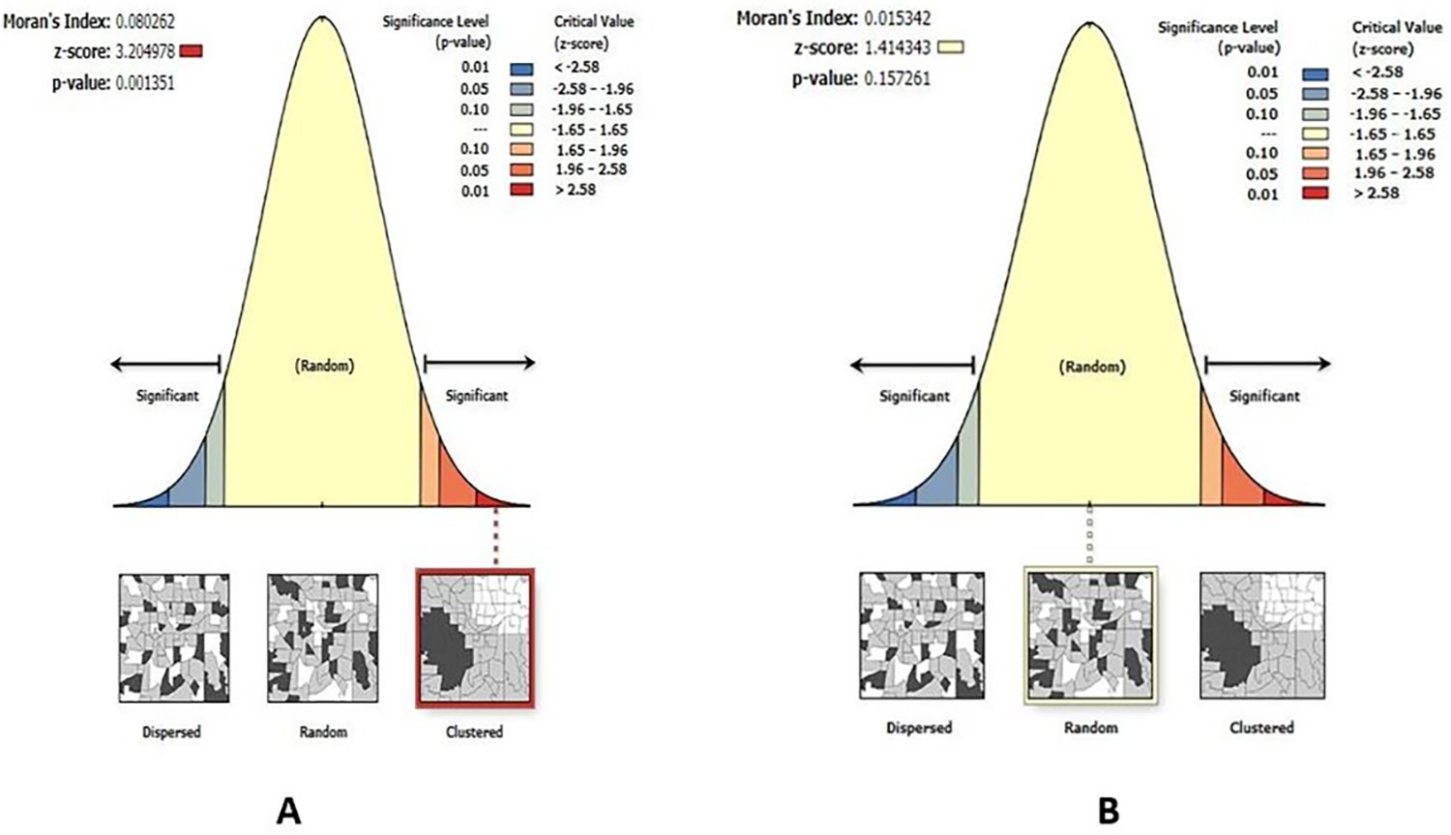
Spatial autocorrelation analysis of OLS (A) and GWR residuals (B) to identify spatial predictors of inadequate meal frequency in Ethiopia, mini EDHS 2019.

**Geographic weighted regression analysis results:** The OLS regression’s findings were untrustworthy due to inconsistent spatial relationships between inadequate meal frequency and independent variables. Additionally, the OLS model residuals were not normally distributed. Hence, GWR model was required to account for the spatial autocorrelation of residuals and inconsistent relationships across space and map area-specific coefficients of independent variables. During GWR analysis, independent variables used to build the OLS model were used. Improvement was achieved in the GWR model as compared to OLS analysis. The adjusted-R^2^ was increased from 19.9% to 24.15%. Which indicates that an additional 4.25% of the spatial variation in inadequate meal frequency was explained by the GWR model. Additionally, the GWR’s AICc value was lower than the OLS’s AICc value (difference = 7.406). The spatial distribution of residuals from the two models were compared. The OLS model residuals were clustered whereas the GWR model residuals were randomly distributed (**Fig 9**). So, the GWR model adequately accounted the spatial heterogeneity in the relationship between independent variables and inadequate meal frequency (**Table 5**).

**Table 5:** Summary of OLS results, model diagnostics and spatial regression model comparison.

Variables	Coefficient	Robust standard error	Robust t-statistic	Robust p-value	VIF
Intercept	0.062	0.080	0.773	0.440	…
Proportion of children aged 6–11 months	0.064	0.079	0.803	0.423	1.724
Proportion of children aged 12–17 months	0.061	0.085	0.725	0.469	1.601
Proportion of children from households headed by males	0.076	0.059	1.280	0.202	1.043
Proportion of children with no timely initiation of breastfeeding	0.025	0.075	0.333	0.739	1.072
Proportion of children who weren’t on breastfeeding	0.222	0.089	2.497	0.013	1.209
Proportion of children born from mothers who hadn’t antenatal care visit (zero ANC)	0.254	0.065	3.927	< 0.001	1.475
Proportion of children born from mothers who had 1 to 3 (inclusive) antenatal care visits	0.026	0.075	0.335	0.456	1.106
Proportion of children born from mothers who hadn’t education	0.187	0.052	3.575	< 0.001	1.335
**OLS model diagnostics**					
**Diagnostic parameters**		**Value**		**P-value**	
Adjusted-R^2^		0.199		…	
AICc		46.190		…	
Joint F-statistic		10.170		< 0.001	
Joint Wald statistic		98.522		< 0.001	
Koenker (BP) statistic		49.308		< 0.001	
Jarque- Bera statistic		69.125		< 0.001	
**Comparison between spatial regression models**					
**Parameter**		**Model**			
		**OLS**		**GWR**	
Adjusted-R^2^		0.199		24.146	
AICc		46.190		38.784	
Distribution of residuals		Clustered (Moran’s I = 0.080, p-value = 0.001)		Random (Moran’s I = 0.015, p-value = 0.157)	

### Spatially varying predictors of inadequate meal frequency

Hotspot areas of inadequate meal frequency were significantly and positively associated with; proportion of children who weren’t on breastfeeding during the survey, proportion of children born from mothers who hadn’t antenatal care visit (zero ANC), and proportion of children born from mothers who hadn’t formal education.

**Fig 10:**
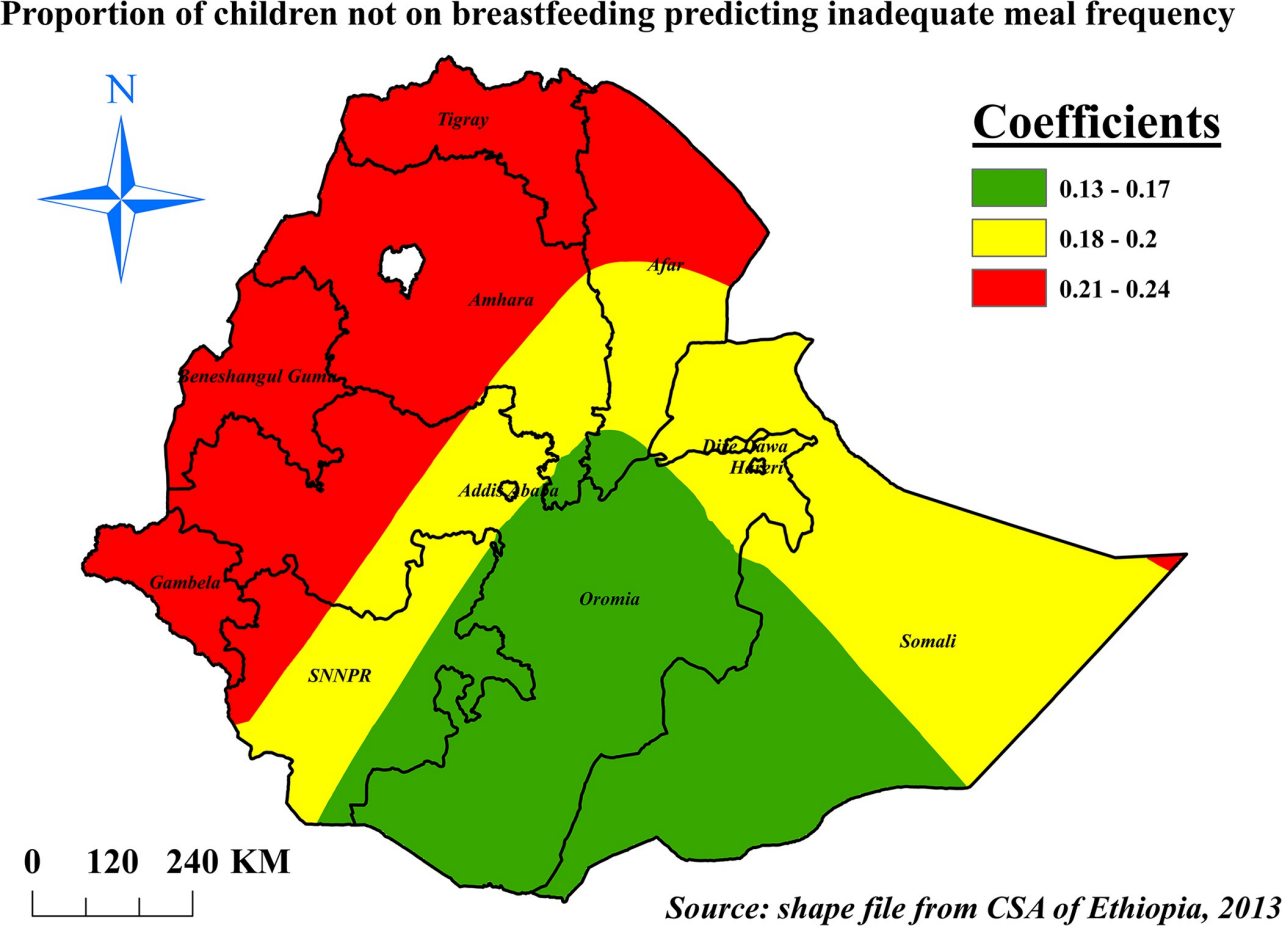
The GWR coefficients of non-breastfeeding children predicting inadequate meal frequency among children aged 6–23 months in Ethiopia, mini EDHS 2019.

The proportion of children who weren’t on breastfeeding during the interview and inadequate meal frequency hotspot areas were found positively associated. The prevalence of inadequate meal frequency was increased by 21% to 24% when the prevalence of non-breastfeeding children increase by 1% in Tigray, Gambela, Beneshangul-Gumuz, northern Afar, western Oromia and SNNPR, and northwest Amhara (**Fig 10**).

**Fig 11:**
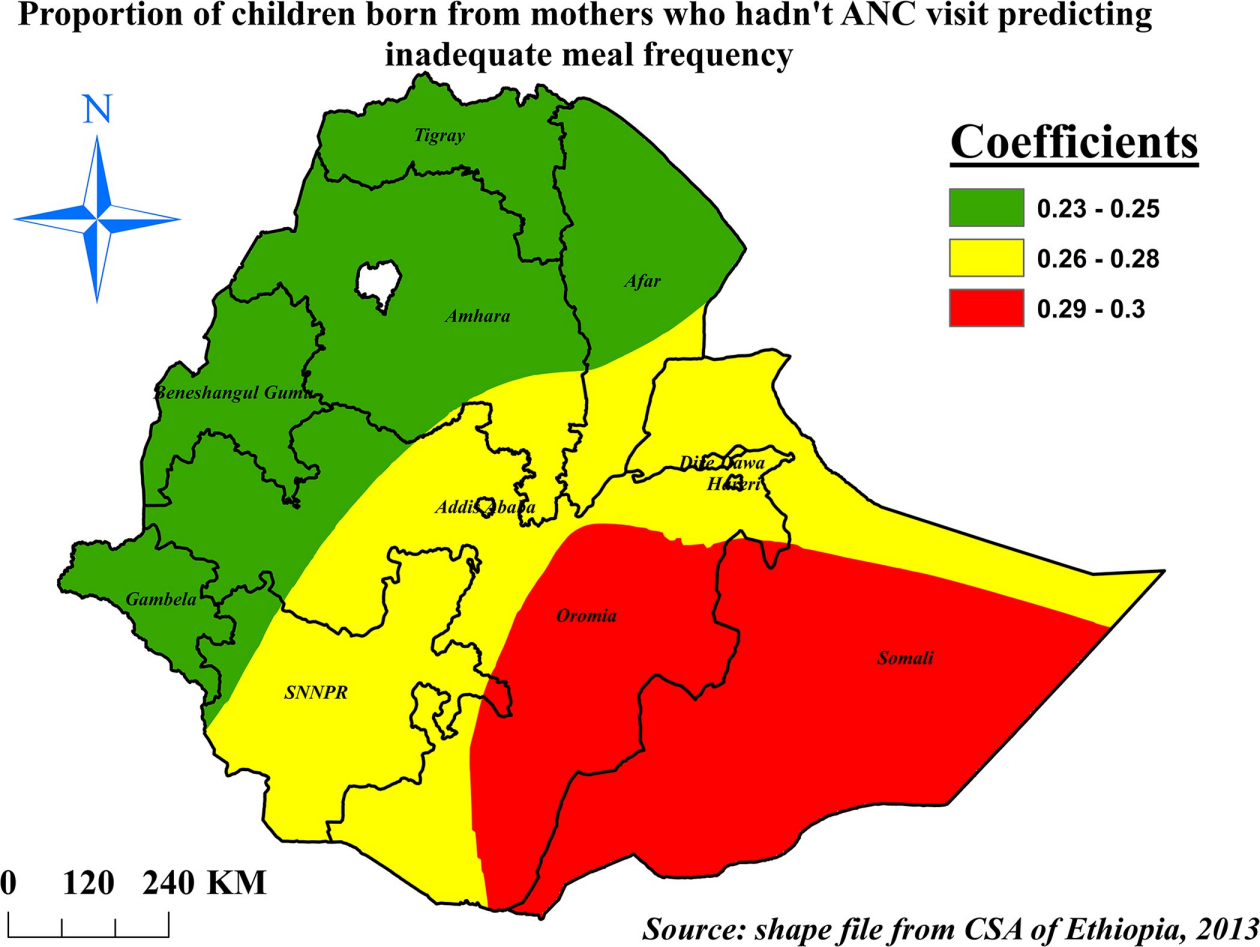
The GWR coefficients of children born from mothers who hadn’t ANC visit predicting inadequate meal frequency among children aged 6–23 months in Ethiopia, mini EDHS 2019.

The percentage of inadequate meal frequency and prevalence of children born from mothers who hadn’t ANC visit were spatially associated significantly. The prevalence of inadequate meal frequency was highly increased (29% to 30%) in southern Somali and Eastern Oromia as the percentage of children born from mothers who hadn’t ANC visit increased by one unit (**Fig 11**).

**Fig 12:**
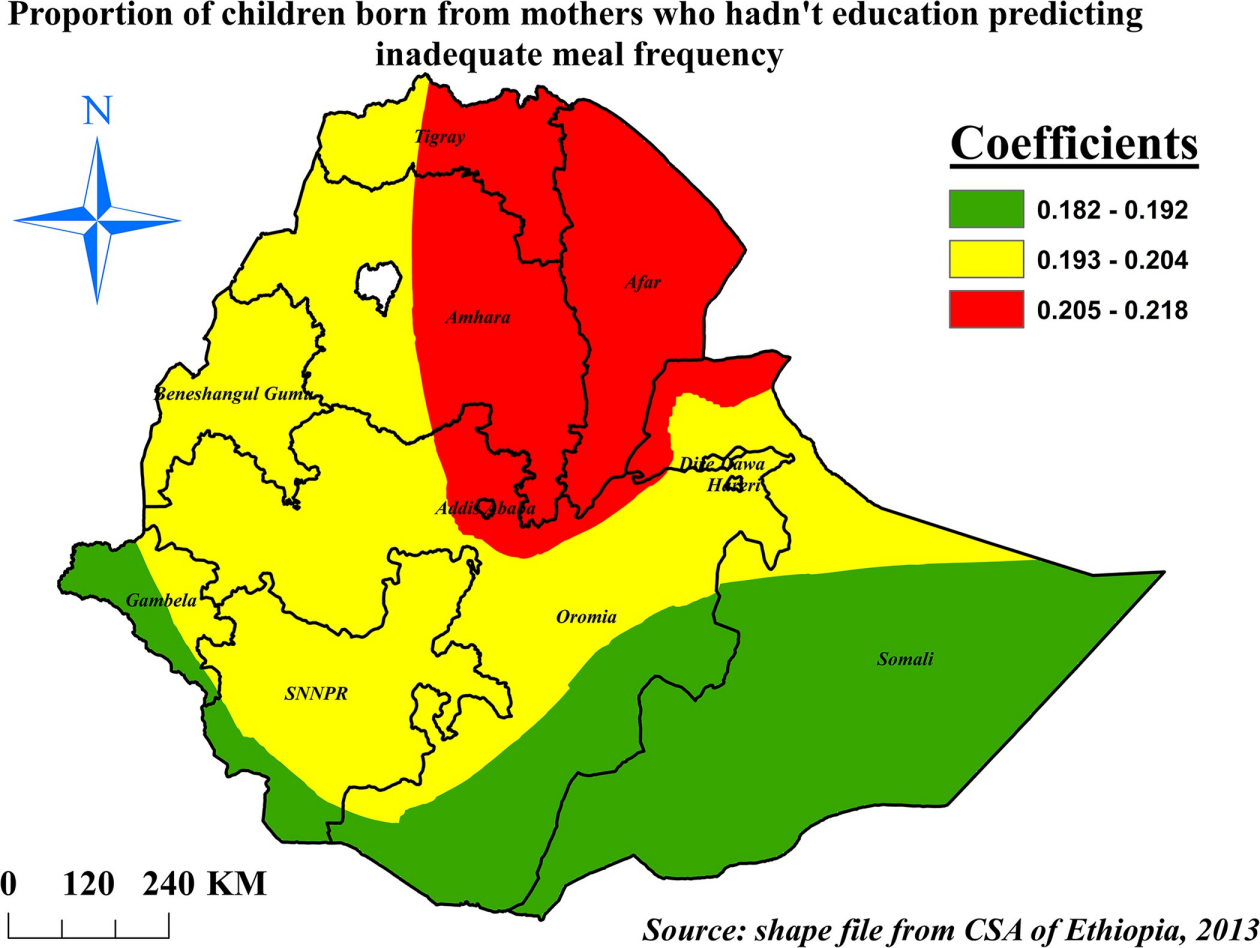
The GWR coefficients of children born from mothers who hadn’t education predicting inadequate meal frequency among children aged 6–23 months in Ethiopia, mini EDHS 2019.

The percentage of children born from mothers who hadn’t education was positively correlated with prevalence of inadequate meal frequency. For a unit increase in the percentage of women who hadn’t education, the prevalence of inadequate meal frequency was increased by 20.5% to 21.8% in eastern Tigray and Amhara, Afar, and Addis Ababa (**Fig 12**).

## Discussion

Inadequate meal frequency during early childhood increase the risk of undernutrition, illness, death and developmental delays in children aged 6–23 months. Evidence based early and appropriate interventions to improve child feeding practices are essential to reduce undernutrition and its consequences among these children. Understanding of the level of meal frequency and its distribution at national level is important for area-targeted interventions in resource limited settings like Ethiopia. Therefore, this study aimed to determine inadequate meal frequency feeding practice, associated factors and its spatial variation among children aged 6–23 months in Ethiopia.

Accordingly, this study showed that the prevalence of inadequate meal frequency among children aged 6–23 months in Ethiopia was 47.03% (95% CI: 44.54%, 49.53%). The prevalence of inadequate meal frequency in Ethiopia was found lower than the findings of a study from Ghana [51], Bangladesh [2], India [17], Nigeria [52], and Burkina Faso [53] that was 56.7%, 52.6%, 58.5%, 66.4%, and 75.6%, respectively. This might be due to time difference among these studies, according to a study from Nepal IYCF practices are improving through time [54]. In addition, this difference might be resulted from heterogeneity across countries in cultural beliefs regarding child feeding practice, sociodemographic and economic dissimilarity of the study population, and nutritional intervention policies [16].

This study finding was also higher than those of studies conducted in Bale zone [5] and Wolaita Sodo town [35] which was 31.6% and 31.1%, respectively. Our study included study subjects across nine regions and two self-administrative cities, areas with high prevalence of inadequate meal frequency might overestimate the national prevalence of inadequate meal frequency. Our study finding was consistent with a study from Gambia. Among Gambian children aged 6–23 months, 57.95% of them achieve MMF (42.05% were had inadequate meal frequency based on UNICEF’s minimum meal frequency criteria) [16]. The two studies were based on the national surveys conducted in 2019, which might result consistent findings on prevalence.

Factors including child age, sex of household head, timely initiation of breastfeeding, current breastfeeding status, number of ANC visit, maternal education, and region were significantly associated with inadequate meal frequency.

Children aged 6–11 and 12–17 months were more risky for inadequate meal frequency as compared to children aged 18–23 months. A research from Gambia indicates 20% and 9% of inadequate meal frequency was attributed to children aged 6–11 and 12–23 months, respectively [55]. Additionally, our study finding is also in line with different studies conducted in Ethiopia [5,6,35], India [17], Sri Lanka [56], Gambia [16], Bangladesh [57] and Ghana [19]. All these studies indicates that the lower the age of the child, the higher the risk of inadequate meal frequency (less likely to achieve the minimum meal frequency requirement). This might be due to that older children are more likely to request for food from their family which helps them to reach minimum daily meal frequency as compared to youngest children [19]. Additionally, this might be also occurred due to that small percentage children aged 6–8 months were introduced for solid, semi-solid or soft food in many countries [57,58]. This is because of that mothers think that lower age children will face difficulty to eat food and it will cause health problems, hence they mayn’t start feeding them in early months of age. This might increase the risk of inadequate daily meal frequency in those age groups. As children get older, they usually leave their mothers breast milk, so have better chance of eating foods frequently.

Children who didn’t started breastfeeding timely (within one hour) after birth were had higher odds of inadequate meal frequency. This finding is consistent with a study conducted in Afar region, Amibara district [27], in which those who didn’t initiated breastfeeding were had lower probability of achieving MMF requirements. This might be due to that mothers who didn’t initiate breastfeeding on time are more likely to continue their inappropriate feeding practice including inadequate meal frequency. Moreover, mothers who didn’t initiate timely breastfeeding for their child are thought to have a previous exposure of inappropriate child feeding practices.

Children from male headed households were had higher odds of inadequate meal frequency as compared to children from female headed households. This finding is supported by a study that assess complementary feeding practices and associated factors in SSA countries [25], northwest Ethiopia [59], and Kenya [60] in which the odds of inadequate meal frequency feeding practices were higher among male household heads compared with their counterparts. This might be attributed to the fact that mothers’ involvement in decision making process regarding main household activities are lower in households headed by males. This might prohibit them in buying necessary food for their children which in turn plays role for inadequate meal frequency. The study from Kenya showed that mothers’ decision making on how to use household income was found positively associated with more appropriate child feeding practices than paternal decisions on household income. This evidence is also corroborated by a study from Bangladesh [2].

Children currently (within 24 hours prior to the interview) not on breastfeeding were had higher odds of inadequate meal frequency. This finding is in line with a study in SSA [25], northwest Ethiopia [27]. This might be due to the difference in the meal frequency requirement among breastfeeding and non-breastfeeding children, since meal frequency requirement is decreased among breastfeeding children. In non-breastfeeding children at least four meals are required per day whereas it decreased in to three meals for breastfeeding children. This might increase the odds of inadequate meal frequency among non-breastfeeding children [61]. This finding is also supported by the GWR analysis results of this study in which being not on breastfeeding were had higher percentages of inadequate meal frequency.

The lower the number of maternal ANC visits, the higher odds of inadequate meal frequency was observed among children in this study. This finding is supported by a study conducted in Shashemene [22], comparison study conducted in India and Sri Lanka [62], This might be due to the effect of counseling provided by health professionals during pregnancy (antenatal care follow-up) on child feeding practice, which increase when the mother had frequent visits [63]. This could imply that promoting ANC visit and a stronger integration with IYCF counselling supports the improvement of infant feeding practices. Based on the evidence at our hand, in the spatial regression analysis children born from mothers who had zero ANC visit were had higher percentages of inadequate meal frequency with varying influence across places.

In this study, children born from mothers who hadn’t education were had higher odds of inadequate meal frequency as compared to children born from mothers who had higher educational status. This finding is supported by previous studies conducted in Ghana [19], Bangladesh [2], south Asia [29], Pakistan [64] and southern Ethiopia [21]. This might be due to that non-educated mothers have difficulties in reading information sources and less exposure to IYCF practice promotions through mass media than educated mothers. Besides, the impact of education in improving maternal knowledge regarding appropriate feeding practice might result better IYCF practices. Hence non-educated mothers usually have lower understanding of nutrition education than educated mothers. This evidence is supported by a study from Nepal [65] and slum areas Bahir Dar city [66]. From the spatial regression analysis children born from non-educated mothers were had higher percentages of inadequate meal frequency with spatially varying influence.

Region was another important factor associated with inadequate meal frequency. Children who reside in Amhara, SNNPR and Gambela were had higher odds of inadequate meal frequency as compared to children residing in Addis Ababa. Based on the evidence at our hand, regional variations of inadequate meal frequency was observed in the spatial analysis that might result higher and lower odds of inadequate meal frequency across regions. From the spatial distribution map, high prevalence of inadequate meal frequency was observed in Amhara, SNNPR and Gambela as compared to that of Addis Ababa. The LISA finding also corroborate this finding in which low-low clusters were observed in Addis Ababa. In the spatial regression analysis (GWR) findings of this study, spatially varying influences of ANC visit, maternal education and child breastfeeding status on inadequate meal frequency may result regional differences. In addition, Addis Ababa is the capital city of Ethiopia, hence mothers who reside there might have better; ANC visits that expose them for appropriate IYCF practice counselling and education which might result lower odds of inadequate meal frequency as compared to other regions. From a previous study in Ethiopia, optimal ANC visits were observed in Addis Ababa whereas it was low in Amhara, Gambela and SNNPR [67]. Another study from Ethiopia also indicates that better maternal health service utilization can improve appropriate child feeding practices [33]. In addition, studies from Kenya [68], and Benin [69] reported the influence of culture on child feeding practice. A qualitative study from northwest Ethiopia explore the relationship between culture and child feeding practices [70]. Additional evidence from a study conducted on dietary behavior and sociocultural determinants of feeding practices in Amhara region supports the influence of culture on feeding practices [71].

The spatial distribution of inadequate meal frequency was showed significant variation across Ethiopia. Significant hotspot areas of inadequate meal frequency were identified in Somali, northern Afar, Harari, Amhara, Gambela and eastern SNNPR. Somali and Harari. The variation of inadequate meal frequency across areas of Ethiopia might be due to the socio-cultural and economical disparities in the country. Additionally, this could be also attributed to the regional disparity in maternal and child health services [72]. A study conducted in Bangladesh also showed significant variation of meal frequency feeding practices across places [2]. This finding highlights the importance of implementing area-specific interventions targeting identified hotspot areas of inadequate meal frequency in Ethiopia to improve infant and young child meal frequency.

### Policy implications

This study has policy implications towards improving IYCF practices. Based on the identified high-risk areas of inadequate meal frequency, resources may be allocated equitably in which more resources may be allocated for hotspot (high prevalence) areas. In addition, context specific interventions might be designed for each hotspot areas taking the identified spatially varying covariates in to account.

### Strength and limitations of the study

Starting from its strength, this study was based on nationally representative data which makes findings to be generalizable for all infant and young children aged 6–23 months. Whereas this study has limitations including; the feeding history of the child was assessed through a 24-hour recall which might be prone to recall bias in which mothers might be failed to remember their child eating frequency in the last 24 hours. In addition, the study might be prone to social desirability bias in which the mother might respond incorrectly in the way that favors her social acceptance. Moreover, due to the cross-sectional nature of the data temporal relationship can’t be established. Furthermore, important variables like media exposure, maternal decision making on household activities, working status, and cultural factors were not included in the analysis because of using secondary data. These factors weren’t available in the 2019 mini EDHS data.

## Conclusion and recommendations

Inadequate meal frequency is high in Ethiopia as compared to WHO recommendation. This study make it very evident that nearly half of Ethiopian infants and young children didn’t achieve the minimum daily meal frequency requirement, which is one of the key complementary feeding indicators. Inadequate meal frequency was found associated with age of the child, sex of the household head, timely initiation of breastfeeding, current breastfeeding status, number of ANC visits, maternal education, and region. Besides, it had significant clustering pattern across the country. Spatially varying significant factors identified were child breastfeeding status, maternal education and ANC visits. Therefore it would be better if policy makers design hotspot area-targeted interventions to reduce the national prevalence of inadequate meal frequency. Enhancing breastfeeding practices, improving number of ANC visits, maternal empowerments through education, should target hotspot areas of inadequate meal frequency to minimize inappropriate feeding practice in the country. Future researches should focus on investigating cultural and behavioral factors associated with child feeding practices in the country.

## Abbreviations

AOR: Adjusted Odds Ratio
CI: : Confidence Interval
EDHS: Ethiopian Demographic and Health Survey
CSA: Central Statistical Agency
ICC: Intra-class Correlation Coefficient
IYCF: Infant and Young Child Feeding
LISA: Local Indicator of Spatial Autocorrelation
LLR: Log-Likelihood Ratio
LL: Log-Likelihood
MOR: Median Odds Ratio
MMF: Minimum Meal Frequency
PCV: Proportional change in variance
VIF: Variance Inflation Factor
WHO: World Health Organization
